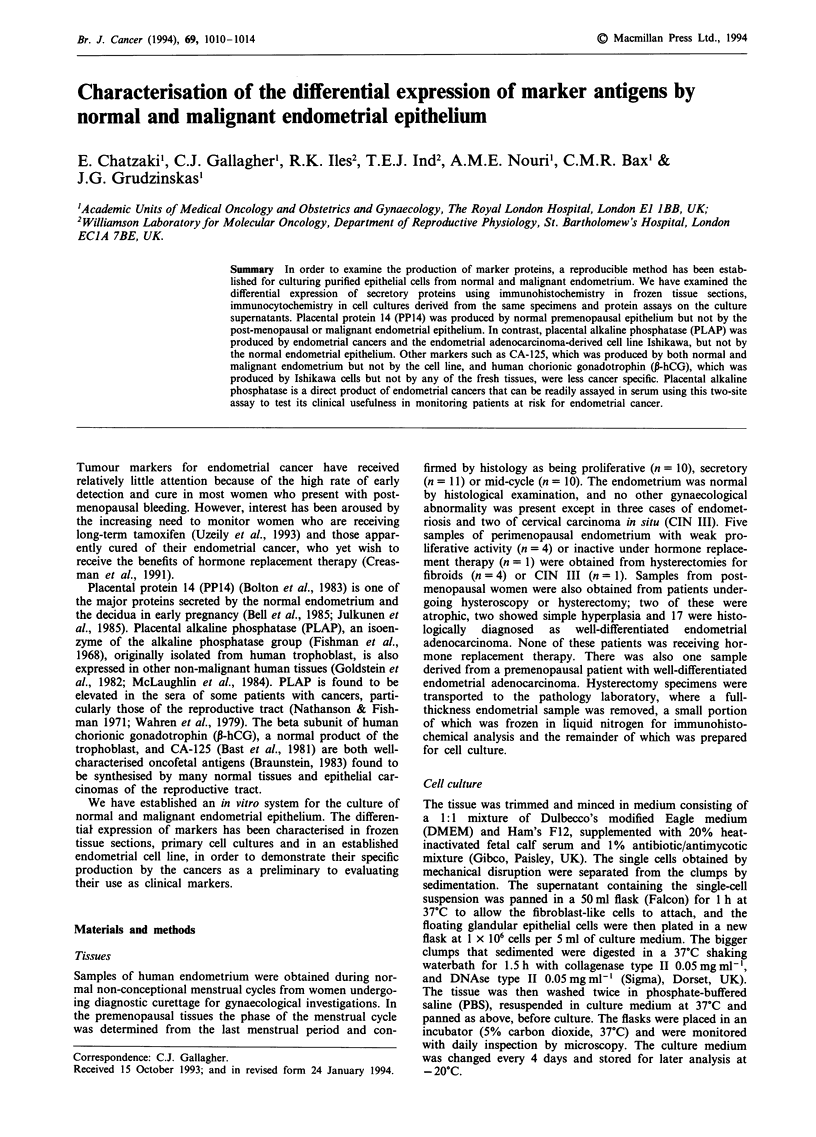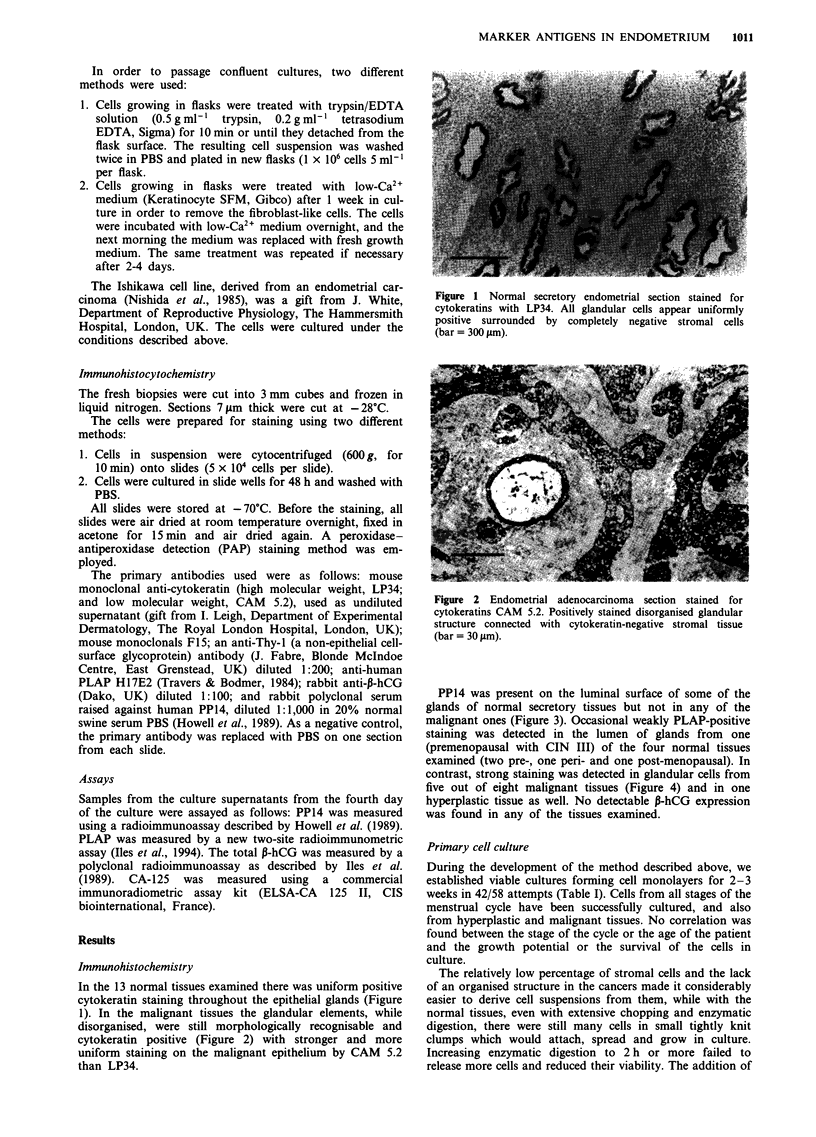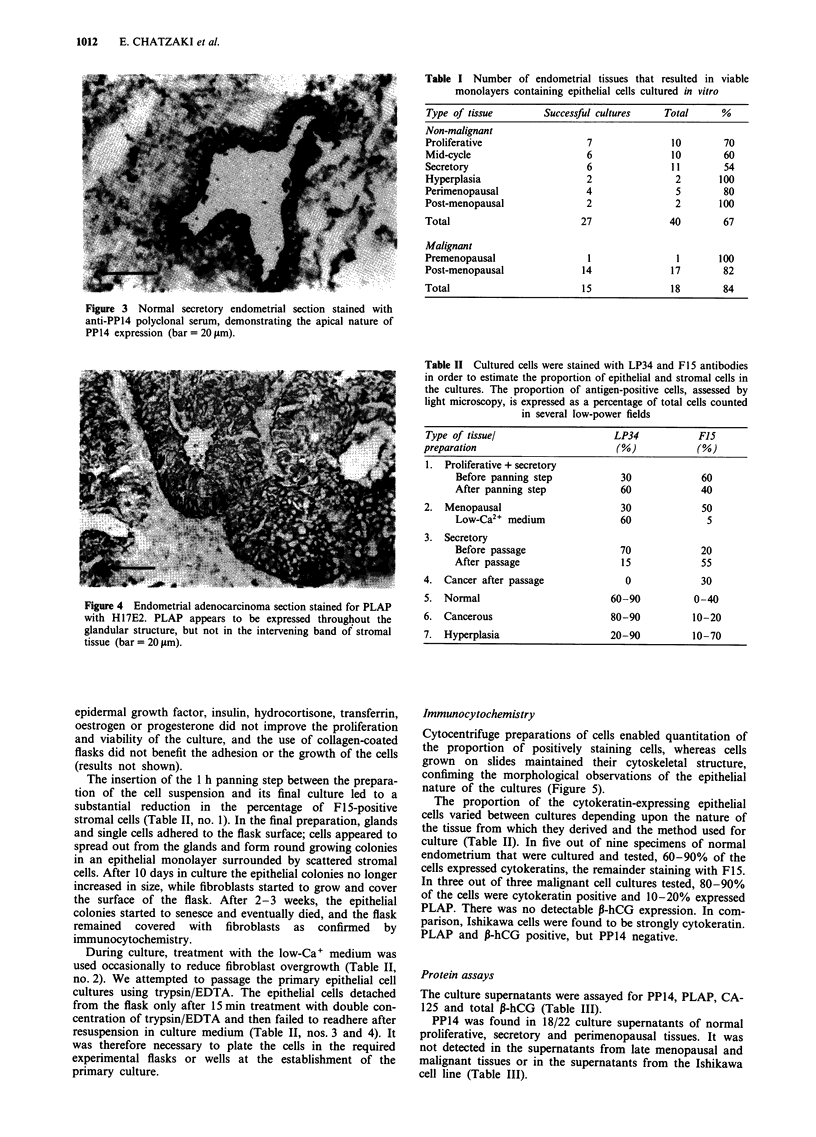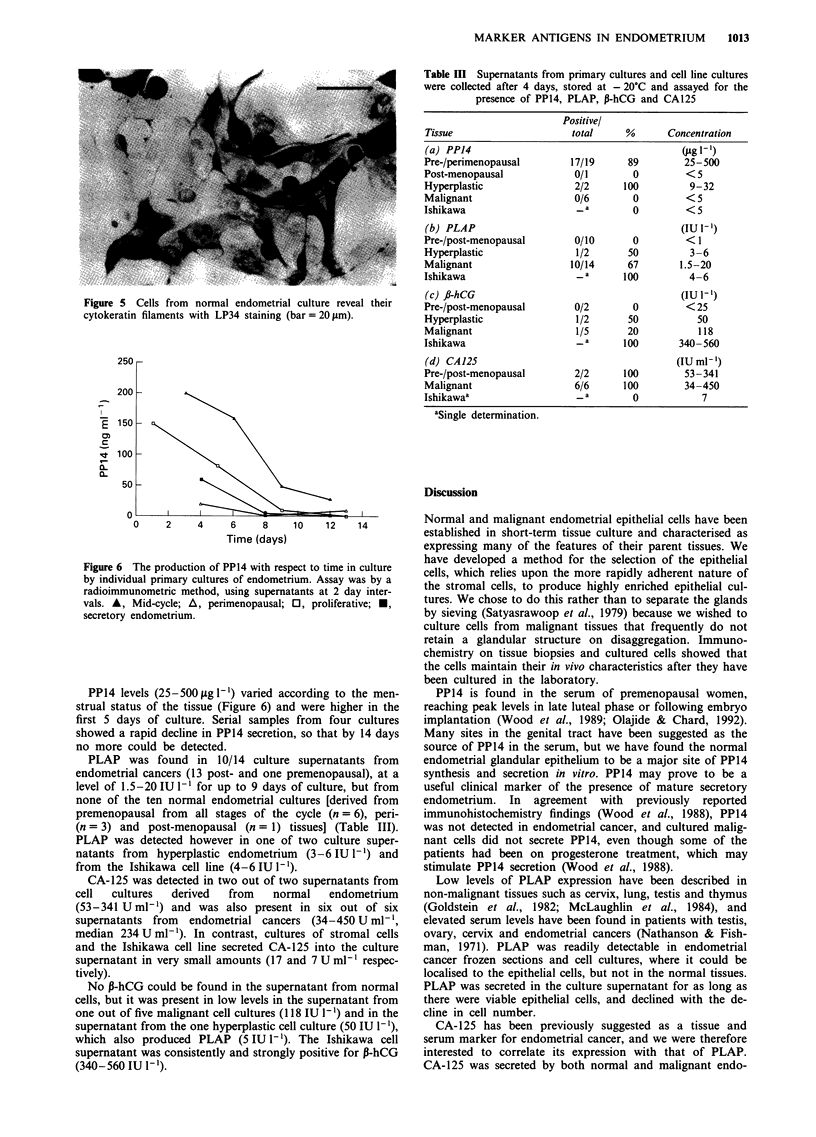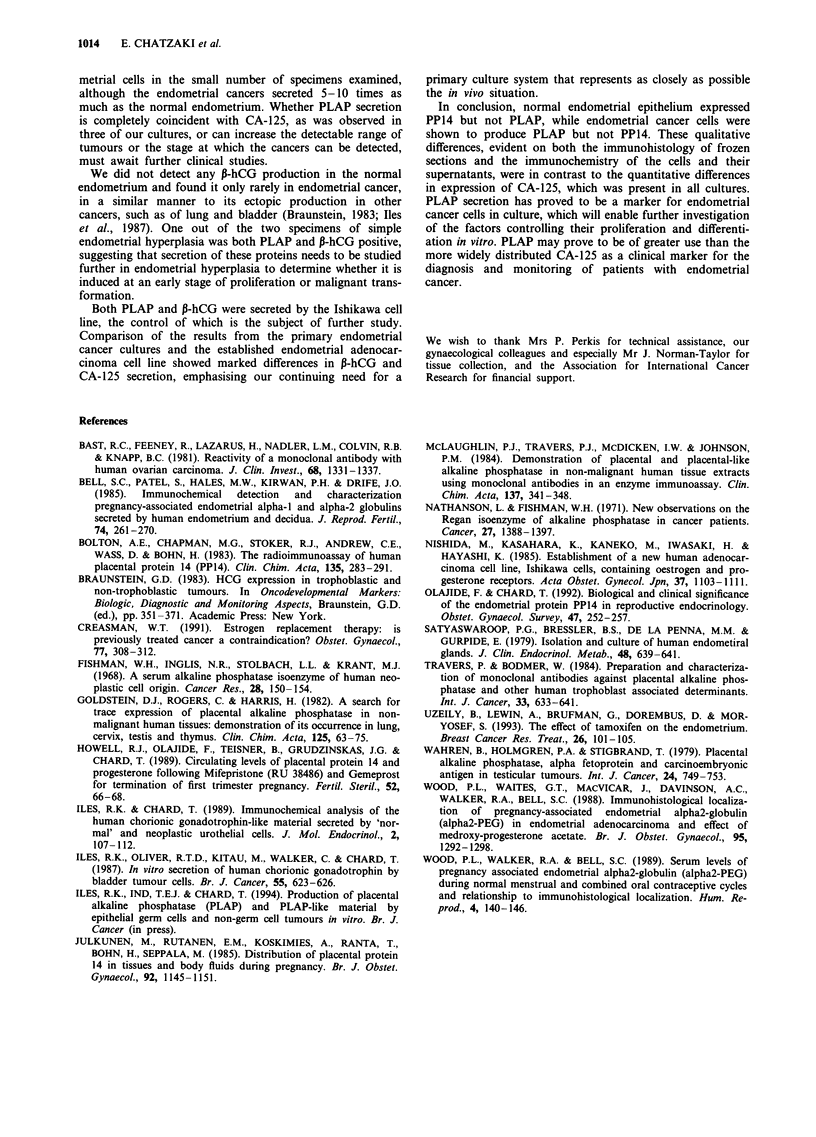# Characterisation of the differential expression of marker antigens by normal and malignant endometrial epithelium.

**DOI:** 10.1038/bjc.1994.198

**Published:** 1994-06

**Authors:** E. Chatzaki, C. J. Gallagher, R. K. Iles, T. E. Ind, A. M. Nouri, C. M. Bax, J. G. Grudzinskas

**Affiliations:** Academic Unit of Medical Oncology, Royal London Hospital, UK.

## Abstract

**Images:**


Br. J. Cancer (1994), 69, 1010-1014 © Macmillan Press Ltd., 1994

### Characterisation of the differential expression of marker antigens by normal and malignant endometrial epithelium

E. Chatzaki[1], C.J. Gallagher[1], R.K. Iles[2], T.E.J. Ind[2], A.M.E. Nouri[1], C.M.R. Bax[1] & J.G. Grudzinskas[1]

[1]*Academic Units of Medical Oncology and Obstetrics and Gynaecology, The Royal London Hospital, London E1 1BB, UK;* [2]*Williamson Laboratory for Molecular Oncology, Department of Reproductive Physiology, St. Bartholomew's Hospital, London EC1A 7BE, UK.*

**Summary** In order to examine the production of marker proteins, a reproducible method has been established for culturing purified epithelial cells from normal and malignant endometrium. We have examined the differential expression of secretory proteins using immunohistochemistry in frozen tissue sections, immunocytochemistry in cell cultures derived from the same specimens and protein assays on the culture supernatants. Placental protein 14 (PP14) was produced by normal premenopausal epithelium but not by the post-menopausal or malignant endometrial epithelium. In contrast, placental alkaline phosphatase (PLAP) was produced by endometrial cancers and the endometrial adenocarcinoma-derived cell line Ishikawa, but not by the normal endometrial epithelium. Other markers such as CA-125, which was produced by both normal and malignant endometrium but not by the cell line, and human chorionic gonadotrophin (β-hCG), which was produced by Ishikawa cells but not by any of the fresh tissues, were less cancer specific. Placental alkaline phosphatase is a direct product of endometrial cancers that can be readily assayed in serum using this two-site assay to test its clinical usefulness in monitoring patients at risk for endometrial cancer.

Tumour markers for endometrial cancer have received relatively little attention because of the high rate of early detection and cure in most women who present with post-menopausal bleeding. However, interest has been aroused by the increasing need to monitor women who are receiving long-term tamoxifen (Uzeily *et al.*, 1993) and those apparently cured of their endometrial cancer, who yet wish to receive the benefits of hormone replacement therapy (Creasman *et al.*, 1991).

Placental protein 14 (PP14) (Bolton *et al.*, 1983) is one of the major proteins secreted by the normal endometrium and the decidua in early pregnancy (Bell *et al.*, 1985; Julkunen *et al.*, 1985). Placental alkaline phosphatase (PLAP), an isoenzyme of the alkaline phosphatase group (Fishman *et al.*, 1968), originally isolated from human trophoblast, is also expressed in other non-malignant human tissues (Goldstein *et al.*, 1982; McLaughlin *et al.*, 1984). PLAP is found to be elevated in the sera of some patients with cancers, particularly those of the reproductive tract (Nathanson & Fishman 1971; Wahren *et al.*, 1979). The beta subunit of human chorionic gonadotrophin (β-hCG), a normal product of the trophoblast, and CA-125 (Bast *et al.*, 1981) are both well-characterised oncofetal antigens (Braunstein, 1983) found to be synthesised by many normal tissues and epithelial carcinomas of the reproductive tract.

We have established an *in vitro* system for the culture of normal and malignant endometrial epithelium. The differential expression of markers has been characterised in frozen tissue sections, primary cell cultures and in an established endometrial cell line, in order to demonstrate their specific production by the cancers as a preliminary to evaluating their use as clinical markers.

### Materials and methods

### *Tissues*

Samples of human endometrium were obtained during normal non-conceptional menstrual cycles from women undergoing diagnostic curettage for gynaecological investigations. In the premenopausal tissues the phase of the menstrual cycle was determined from the last menstrual period and confirmed by histology as being proliferative ($n = 10$), secretory ($n = 11$) or mid-cycle ($n = 10$). The endometrium was normal by histological examination, and no other gynaecological abnormality was present except in three cases of endometriosis and two of cervical carcinoma *in situ* (CIN III). Five samples of perimenopausal endometrium with weak proliferative activity ($n = 4$) or inactive under hormone replacement therapy ($n = 1$) were obtained from hysterectomies for fibroids ($n = 4$) or CIN III ($n = 1$). Samples from post-menopausal women were also obtained from patients undergoing hysteroscopy or hysterectomy; two of these were atrophic, two showed simple hyperplasia and 17 were histologically diagnosed as well-differentiated endometrial adenocarcinoma. None of these patients was receiving hormone replacement therapy. There was also one sample derived from a premenopausal patient with well-differentiated endometrial adenocarcinoma. Hysterectomy specimens were transported to the pathology laboratory, where a full-thickness endometrial sample was removed, a small portion of which was frozen in liquid nitrogen for immunohistochemical analysis and the remainder of which was prepared for cell culture.

### *Cell culture*

The tissue was trimmed and minced in medium consisting of a 1:1 mixture of Dulbecco's modified Eagle medium (DMEM) and Ham's F12, supplemented with 20% heat-inactivated fetal calf serum and 1% antibiotic/antimycotic mixture (Gibco, Paisley, UK). The single cells obtained by mechanical disruption were separated from the clumps by sedimentation. The supernatant containing the single-cell suspension was panned in a 50 ml flask (Falcon) for 1 h at 37°C to allow the fibroblast-like cells to attach, and the floating glandular epithelial cells were then plated in a new flask at $1 \times 10^6$ cells per 5 ml of culture medium. The bigger clumps that sedimented were digested in a 37°C shaking waterbath for 1.5 h with collagenase type II 0.05 mg ml$^{-1}$, and DNAse type II 0.05 mg ml$^{-1}$ (Sigma), Dorset, UK). The tissue was then washed twice in phosphate-buffered saline (PBS), resuspended in culture medium at 37°C and panned as above, before culture. The flasks were placed in an incubator (5% carbon dioxide, 37°C) and were monitored with daily inspection by microscopy. The culture medium was changed every 4 days and stored for later analysis at −20°C.

Correspondence: C.J. Gallagher.
Received 15 October 1993; and in revised form 24 January 1994.

In order to passage confluent cultures, two different methods were used:

1. Cells growing in flasks were treated with trypsin/EDTA solution (0.5 g $ml^{-1}$ trypsin, 0.2 g $ml^{-1}$ tetrasodium EDTA, Sigma) for 10 min or until they detached from the flask surface. The resulting cell suspension was washed twice in PBS and plated in new flasks ($1 \times 10^6$ cells 5 $ml^{-1}$ per flask.
2. Cells growing in flasks were treated with low-$Ca^{2+}$ medium (Keratinocyte SFM, Gibco) after 1 week in culture in order to remove the fibroblast-like cells. The cells were incubated with low-$Ca^{2+}$ medium overnight, and the next morning the medium was replaced with fresh growth medium. The same treatment was repeated if necessary after 2-4 days.

The Ishikawa cell line, derived from an endometrial carcinoma (Nishida *et al.*, 1985), was a gift from J. White, Department of Reproductive Physiology, The Hammersmith Hospital, London, UK. The cells were cultured under the conditions described above.

*Immunohistocytochemistry*

The fresh biopsies were cut into 3 mm cubes and frozen in liquid nitrogen. Sections 7 μm thick were cut at −28°C.

The cells were prepared for staining using two different methods:

1. Cells in suspension were cytocentrifuged (600 *g*, for 10 min) onto slides ($5 \times 10^4$ cells per slide).
2. Cells were cultured in slide wells for 48 h and washed with PBS.

All slides were stored at −70°C. Before the staining, all slides were air dried at room temperature overnight, fixed in acetone for 15 min and air dried again. A peroxidase–antiperoxidase detection (PAP) staining method was employed.

The primary antibodies used were as follows: mouse monoclonal anti-cytokeratin (high molecular weight, LP34; and low molecular weight, CAM 5.2), used as undiluted supernatant (gift from I. Leigh, Department of Experimental Dermatology, The Royal London Hospital, London, UK); mouse monoclonals F15; an anti-Thy-1 (a non-epithelial cell-surface glycoprotein) antibody (J. Fabre, Blonde McIndoe Centre, East Grenstead, UK) diluted 1:200; anti-human PLAP H17E2 (Travers & Bodmer, 1984); rabbit anti-β-hCG (Dako, UK) diluted 1:100; and rabbit polyclonal serum raised against human PP14, diluted 1:1,000 in 20% normal swine serum PBS (Howell *et al.*, 1989). As a negative control, the primary antibody was replaced with PBS on one section from each slide.

*Assays*

Samples from the culture supernatants from the fourth day of the culture were assayed as follows: PP14 was measured using a radioimmunoassay described by Howell *et al.* (1989). PLAP was measured by a new two-site radioimmunometric assay (Iles *et al.*, 1994). The total β-hCG was measured by a polyclonal radioimmunoassay as described by Iles *et al.* (1989). CA-125 was measured using a commercial immunoradiometric assay kit (ELSA-CA 125 II, CIS biointernational, France).

### Results

*Immunohistochemistry*

In the 13 normal tissues examined there was uniform positive cytokeratin staining throughout the epithelial glands (Figure 1). In the malignant tissues the glandular elements, while disorganised, were still morphologically recognisable and cytokeratin positive (Figure 2) with stronger and more uniform staining on the malignant epithelium by CAM 5.2 than LP34.

**Figure 1** Normal secretory endometrial section stained for cytokeratins with LP34. All glandular cells appear uniformly positive surrounded by completely negative stromal cells (bar = 300 μm).

**Figure 2** Endometrial adenocarcinoma section stained for cytokeratins CAM 5.2. Positively stained disorganised glandular structure connected with cytokeratin-negative stromal tissue (bar = 30 μm).

PP14 was present on the luminal surface of some of the glands of normal secretory tissues but not in any of the malignant ones (Figure 3). Occasional weakly PLAP-positive staining was detected in the lumen of glands from one (premenopausal with CIN III) of the four normal tissues examined (two pre-, one peri- and one post-menopausal). In contrast, strong staining was detected in glandular cells from five out of eight malignant tissues (Figure 4) and in one hyperplastic tissue as well. No detectable β-hCG expression was found in any of the tissues examined.

*Primary cell culture*

During the development of the method described above, we established viable cultures forming cell monolayers for 2–3 weeks in 42/58 attempts (Table I). Cells from all stages of the menstrual cycle have been successfully cultured, and also from hyperplastic and malignant tissues. No correlation was found between the stage of the cycle or the age of the patient and the growth potential or the survival of the cells in culture.

The relatively low percentage of stromal cells and the lack of an organised structure in the cancers made it considerably easier to derive cell suspensions from them, while with the normal tissues, even with extensive chopping and enzymatic digestion, there were still many cells in small tightly knit clumps which would attach, spread and grow in culture. Increasing enzymatic digestion to 2 h or more failed to release more cells and reduced their viability. The addition of

**Figure 3** Normal secretory endometrial section stained with anti-PP14 polyclonal serum, demonstrating the apical nature of PP14 expression (bar = 20 μm).

**Figure 4** Endometrial adenocarcinoma section stained for PLAP with H17E2. PLAP appears to be expressed throughout the glandular structure, but not in the intervening band of stromal tissue (bar = 20 μm).

**Table I** Number of endometrial tissues that resulted in viable monolayers containing epithelial cells cultured *in vitro*

| *Type of tissue* | *Successful cultures* | *Total* | *%* |
|---|---|---|---|
| *Non-malignant* | | | |
| Proliferative | 7 | 10 | 70 |
| Mid-cycle | 6 | 10 | 60 |
| Secretory | 6 | 11 | 54 |
| Hyperplasia | 2 | 2 | 100 |
| Perimenopausal | 4 | 5 | 80 |
| Post-menopausal | 2 | 2 | 100 |
| Total | 27 | 40 | 67 |
| *Malignant* | | | |
| Premenopausal | 1 | 1 | 100 |
| Post-menopausal | 14 | 17 | 82 |
| Total | 15 | 18 | 84 |

**Table II** Cultured cells were stained with LP34 and F15 antibodies in order to estimate the proportion of epithelial and stromal cells in the cultures. The proportion of antigen-positive cells, assessed by light microscopy, is expressed as a percentage of total cells counted in several low-power fields

| *Type of tissue/ preparation* | *LP34 (%)* | *F15 (%)* |
|---|---|---|
| 1. Proliferative + secretory | | |
| Before panning step | 30 | 60 |
| After panning step | 60 | 40 |
| 2. Menopausal | 30 | 50 |
| Low-$Ca^{2+}$ medium | 60 | 5 |
| 3. Secretory | | |
| Before passage | 70 | 20 |
| After passage | 15 | 55 |
| 4. Cancer after passage | 0 | 30 |
| 5. Normal | 60-90 | 0-40 |
| 6. Cancerous | 80-90 | 10-20 |
| 7. Hyperplasia | 20-90 | 10-70 |

epidermal growth factor, insulin, hydrocortisone, transferrin, oestrogen or progesterone did not improve the proliferation and viability of the culture, and the use of collagen-coated flasks did not benefit the adhesion or the growth of the cells (results not shown).

The insertion of the 1 h panning step between the preparation of the cell suspension and its final culture led to a substantial reduction in the percentage of F15-positive stromal cells (Table II, no. 1). In the final preparation, glands and single cells adhered to the flask surface; cells appeared to spread out from the glands and form round growing colonies in an epithelial monolayer surrounded by scattered stromal cells. After 10 days in culture the epithelial colonies no longer increased in size, while fibroblasts started to grow and cover the surface of the flask. After 2-3 weeks, the epithelial colonies started to senesce and eventually died, and the flask remained covered with fibroblasts as confirmed by immunocytochemistry.

During culture, treatment with the low-$Ca^{+}$ medium was used occasionally to reduce fibroblast overgrowth (Table II, no. 2). We attempted to passage the primary epithelial cell cultures using trypsin/EDTA. The epithelial cells detached from the flask only after 15 min treatment with double concentration of trypsin/EDTA and then failed to readhere after resuspension in culture medium (Table II, nos. 3 and 4). It was therefore necessary to plate the cells in the required experimental flasks or wells at the establishment of the primary culture.

*Immunocytochemistry*

Cytocentrifuge preparations of cells enabled quantitation of the proportion of positively staining cells, whereas cells grown on slides maintained their cytoskeletal structure, confiming the morphological observations of the epithelial nature of the cultures (Figure 5).

The proportion of the cytokeratin-expressing epithelial cells varied between cultures depending upon the nature of the tissue from which they derived and the method used for culture (Table II). In five out of nine specimens of normal endometrium that were cultured and tested, 60-90% of the cells expressed cytokeratins, the remainder staining with F15. In three out of three malignant cell cultures tested, 80-90% of the cells were cytokeratin positive and 10-20% expressed PLAP. There was no detectable β-hCG expression. In comparison, Ishikawa cells were found to be strongly cytokeratin. PLAP and β-hCG positive, but PP14 negative.

*Protein assays*

The culture supernatants were assayed for PP14, PLAP, CA-125 and total β-hCG (Table III).

PP14 was found in 18/22 culture supernatants of normal proliferative, secretory and perimenopausal tissues. It was not detected in the supernatants from late menopausal and malignant tissues or in the supernatants from the Ishikawa cell line (Table III).

**Figure 5** Cells from normal endometrial culture reveal their cytokeratin filaments with LP34 staining (bar = 20 μm).

**Figure 6** The production of PP14 with respect to time in culture by individual primary cultures of endometrium. Assay was by a radioimmunometric method, using supernatants at 2 day intervals. ▲, Mid-cycle; Δ, perimenopausal; □, proliferative; ■, secretory endometrium.

PP14 levels (25–500 $\mu g\,l^{-1}$) varied according to the menstrual status of the tissue (Figure 6) and were higher in the first 5 days of culture. Serial samples from four cultures showed a rapid decline in PP14 secretion, so that by 14 days no more could be detected.

PLAP was found in 10/14 culture supernatants from endometrial cancers (13 post- and one premenopausal), at a level of 1.5–20 $IU\,l^{-1}$ for up to 9 days of culture, but from none of the ten normal endometrial cultures [derived from premenopausal from all stages of the cycle ($n = 6$), peri- ($n = 3$) and post-menopausal ($n = 1$) tissues] (Table III). PLAP was detected however in one of two culture supernatants from hyperplastic endometrium (3–6 $IU\,l^{-1}$) and from the Ishikawa cell line (4–6 $IU\,l^{-1}$).

CA-125 was detected in two out of two supernatants from cell cultures derived from normal endometrium (53–341 $U\,ml^{-1}$) and was also present in six out of six supernatants from endometrial cancers (34–450 $U\,ml^{-1}$, median 234 $U\,ml^{-1}$). In contrast, cultures of stromal cells and the Ishikawa cell line secreted CA-125 into the culture supernatant in very small amounts (17 and 7 $U\,ml^{-1}$ respectively).

No β-hCG could be found in the supernatant from normal cells, but it was present in low levels in the supernatant from one out of five malignant cell cultures (118 $IU\,l^{-1}$) and in the supernatant from the one hyperplastic cell culture (50 $IU\,l^{-1}$), which also produced PLAP (5 $IU\,l^{-1}$). The Ishikawa cell supernatant was consistently and strongly positive for β-hCG (340–560 $IU\,l^{-1}$).

**Table III** Supernatants from primary cultures and cell line cultures were collected after 4 days, stored at −20°C and assayed for the presence of PP14, PLAP, β-hCG and CA125

| *Tissue* | *Positive/ total* | *%* | *Concentration* |
|---|---|---|---|
| *(a) PP14* | | | ($\mu g\,l^{-1}$) |
| Pre-/perimenopausal | 17/19 | 89 | 25–500 |
| Post-menopausal | 0/1 | 0 | <5 |
| Hyperplastic | 2/2 | 100 | 9–32 |
| Malignant | 0/6 | 0 | <5 |
| Ishikawa | –[a] | 0 | <5 |
| *(b) PLAP* | | | ($IU\,l^{-1}$) |
| Pre-/post-menopausal | 0/10 | 0 | <1 |
| Hyperplastic | 1/2 | 50 | 3–6 |
| Malignant | 10/14 | 67 | 1.5–20 |
| Ishikawa | –[a] | 100 | 4–6 |
| *(c) β-hCG* | | | ($IU\,l^{-1}$) |
| Pre-/post-menopausal | 0/2 | 0 | <25 |
| Hyperplastic | 1/2 | 50 | 50 |
| Malignant | 1/5 | 20 | 118 |
| Ishikawa | –[a] | 100 | 340–560 |
| *(d) CA125* | | | ($IU\,ml^{-1}$) |
| Pre-/post-menopausal | 2/2 | 100 | 53–341 |
| Malignant | 6/6 | 100 | 34–450 |
| Ishikawa[a] | –[a] | 0 | 7 |

[a]Single determination.

### Discussion

Normal and malignant endometrial epithelial cells have been established in short-term tissue culture and characterised as expressing many of the features of their parent tissues. We have developed a method for the selection of the epithelial cells, which relies upon the more rapidly adherent nature of the stromal cells, to produce highly enriched epithelial cultures. We chose to do this rather than to separate the glands by sieving (Satyasrawoop *et al.*, 1979) because we wished to culture cells from malignant tissues that frequently do not retain a glandular structure on disaggregation. Immunochemistry on tissue biopsies and cultured cells showed that the cells maintain their *in vivo* characteristics after they have been cultured in the laboratory.

PP14 is found in the serum of premenopausal women, reaching peak levels in late luteal phase or following embryo implantation (Wood *et al.*, 1989; Olajide & Chard, 1992). Many sites in the genital tract have been suggested as the source of PP14 in the serum, but we have found the normal endometrial glandular epithelium to be a major site of PP14 synthesis and secretion *in vitro*. PP14 may prove to be a useful clinical marker of the presence of mature secretory endometrium. In agreement with previously reported immunohistochemistry findings (Wood *et al.*, 1988), PP14 was not detected in endometrial cancer, and cultured malignant cells did not secrete PP14, even though some of the patients had been on progesterone treatment, which may stimulate PP14 secretion (Wood *et al.*, 1988).

Low levels of PLAP expression have been described in non-malignant tissues such as cervix, lung, testis and thymus (Goldstein *et al.*, 1982; McLaughlin *et al.*, 1984), and elevated serum levels have been found in patients with testis, ovary, cervix and endometrial cancers (Nathanson & Fishman, 1971). PLAP was readily detectable in endometrial cancer frozen sections and cell cultures, where it could be localised to the epithelial cells, but not in the normal tissues. PLAP was secreted in the culture supernatant for as long as there were viable epithelial cells, and declined with the decline in cell number.

CA-125 has been previously suggested as a tissue and serum marker for endometrial cancer, and we were therefore interested to correlate its expression with that of PLAP. CA-125 was secreted by both normal and malignant endometrial cells in the small number of specimens examined, although the endometrial cancers secreted 5–10 times as much as the normal endometrium. Whether PLAP secretion is completely coincident with CA-125, as was observed in three of our cultures, or can increase the detectable range of tumours or the stage at which the cancers can be detected, must await further clinical studies.

We did not detect any β-hCG production in the normal endometrium and found it only rarely in endometrial cancer, in a similar manner to its ectopic production in other cancers, such as of lung and bladder (Braunstein, 1983; Iles *et al.*, 1987). One out of the two specimens of simple endometrial hyperplasia was both PLAP and β-hCG positive, suggesting that secretion of these proteins needs to be studied further in endometrial hyperplasia to determine whether it is induced at an early stage of proliferation or malignant transformation.

Both PLAP and β-hCG were secreted by the Ishikawa cell line, the control of which is the subject of further study. Comparison of the results from the primary endometrial cancer cultures and the established endometrial adenocarcinoma cell line showed marked differences in β-hCG and CA-125 secretion, emphasising our continuing need for a primary culture system that represents as closely as possible the *in vivo* situation.

In conclusion, normal endometrial epithelium expressed PP14 but not PLAP, while endometrial cancer cells were shown to produce PLAP but not PP14. These qualitative differences, evident on both the immunohistology of frozen sections and the immunochemistry of the cells and their supernatants, were in contrast to the quantitative differences in expression of CA-125, which was present in all cultures. PLAP secretion has proved to be a marker for endometrial cancer cells in culture, which will enable further investigation of the factors controlling their proliferation and differentiation *in vitro*. PLAP may prove to be of greater use than the more widely distributed CA-125 as a clinical marker for the diagnosis and monitoring of patients with endometrial cancer.

We wish to thank Mrs P. Perkis for technical assistance, our gynaecological colleagues and especially Mr J. Norman-Taylor for tissue collection, and the Association for International Cancer Research for financial support.

### References

BAST, R.C., FEENEY, R., LAZARUS, H., NADLER, L.M., COLVIN, R.B. & KNAPP, B.C. (1981). Reactivity of a monoclonal antibody with human ovarian carcinoma. *J. Clin. Invest.*, **68**, 1331–1337.

BELL, S.C., PATEL, S., HALES, M.W., KIRWAN, P.H. & DRIFE, J.O. (1985). Immunochemical detection and characterization pregnancy-associated endometrial alpha-1 and alpha-2 globulins secreted by human endometrium and decidua. *J. Reprod. Fertil.*, **74**, 261–270.

BOLTON, A.E., CHAPMAN, M.G., STOKER, R.J., ANDREW, C.E., WASS, D. & BOHN, H. (1983). The radioimmunoassay of human placental protein 14 (PP14). *Clin. Chim. Acta*, **135**, 283–291.

BRAUNSTEIN, G.D. (1983). HCG expression in trophoblastic and non-trophoblastic tumours. In *Oncodevelopmental Markers: Biologic, Diagnostic and Monitoring Aspects*, Braunstein, G.D. (ed.), pp. 351–371. Academic Press: New York.

CREASMAN, W.T. (1991). Estrogen replacement therapy: is previously treated cancer a contraindication? *Obstet. Gynaecol.*, **77**, 308–312.

FISHMAN, W.H., INGLIS, N.R., STOLBACH, L.L. & KRANT, M.J. (1968). A serum alkaline phosphatase isoenzyme of human neoplastic cell origin. *Cancer Res.*, **28**, 150–154.

GOLDSTEIN, D.J., ROGERS, C. & HARRIS, H. (1982). A search for trace expression of placental alkaline phosphatase in non-malignant human tissues: demonstration of its occurrence in lung, cervix, testis and thymus. *Clin. Chim. Acta*, **125**, 63–75.

HOWELL, R.J., OLAJIDE, F., TEISNER, B., GRUDZINSKAS, J.G. & CHARD, T. (1989). Circulating levels of placental protein 14 and progesterone following Mifepristone (RU 38486) and Gemeprost for termination of first trimester pregnancy. *Fertil. Steril.*, **52**, 66–68.

ILES, R.K. & CHARD, T. (1989). Immunochemical analysis of the human chorionic gonadotrophin-like material secreted by 'normal' and neoplastic urothelial cells. *J. Mol. Endocrinol.*, **2**, 107–112.

ILES, R.K., OLIVER, R.T.D., KITAU, M., WALKER, C. & CHARD, T. (1987). *In vitro* secretion of human chorionic gonadotrophin by bladder tumour cells. *Br. J. Cancer*, **55**, 623–626.

ILES, R.K., IND, T.E.J. & CHARD, T. (1994). Production of placental alkaline phosphatase (PLAP) and PLAP-like material by epithelial germ cells and non-germ cell tumours *in vitro*. *Br. J. Cancer* (in press).

JULKUNEN, M., RUTANEN, E.M., KOSKIMIES, A., RANTA, T., BOHN, H., SEPPALA, M. (1985). Distribution of placental protein 14 in tissues and body fluids during pregnancy. *Br. J. Obstet. Gynaecol.*, **92**, 1145–1151.

McLAUGHLIN, P.J., TRAVERS, P.J., McDICKEN, I.W. & JOHNSON, P.M. (1984). Demonstration of placental and placental-like alkaline phosphatase in non-malignant human tissue extracts using monoclonal antibodies in an enzyme immunoassay. *Clin. Chim. Acta*, **137**, 341–348.

NATHANSON, L. & FISHMAN, W.H. (1971). New observations on the Regan isoenzyme of alkaline phosphatase in cancer patients. *Cancer*, **27**, 1388–1397.

NISHIDA, M., KASAHARA, K., KANEKO, M., IWASAKI, H. & HAYASHI, K. (1985). Establishment of a new human adenocarcinoma cell line, Ishikawa cells, containing oestrogen and progesterone receptors. *Acta Obstet. Gynecol. Jpn*, **37**, 1103–1111.

OLAJIDE, F. & CHARD, T. (1992). Biological and clinical significance of the endometrial protein PP14 in reproductive endocrinology. *Obstet. Gynaecol. Survey*, **47**, 252–257.

SATYASWAROOP, P.G., BRESSLER, B.S., DE LA PENNA, M.M. & GURPIDE, E. (1979). Isolation and culture of human endometiral glands. *J. Clin. Endocrinol. Metab.*, **48**, 639–641.

TRAVERS, P. & BODMER, W. (1984). Preparation and characterization of monoclonal antibodies against placental alkaline phosphatase and other human trophoblast associated determinants. *Int. J. Cancer*, **33**, 633–641.

UZEILY, B., LEWIN, A., BRUFMAN, G., DOREMBUS, D. & MOR-YOSEF, S. (1993). The effect of tamoxifen on the endometrium. *Breast Cancer Res. Treat.*, **26**, 101–105.

WAHREN, B., HOLMGREN, P.A. & STIGBRAND, T. (1979). Placental alkaline phosphatase, alpha fetoprotein and carcinoembryonic antigen in testicular tumours. *Int. J. Cancer*, **24**, 749–753.

WOOD, P.L., WAITES, G.T., MACVICAR, J., DAVINSON, A.C., WALKER, R.A., BELL, S.C. (1988). Immunohistological localization of pregnancy-associated endometrial alpha2-globulin (alpha2-PEG) in endometrial adenocarcinoma and effect of medroxy-progesterone acetate. *Br. J. Obstet. Gynaecol.*, **95**, 1292–1298.

WOOD, P.L., WALKER, R.A. & BELL, S.C. (1989). Serum levels of pregnancy associated endometrial alpha2-globulin (alpha2-PEG) during normal menstrual and combined oral contraceptive cycles and relationship to immunohistological localization. *Hum. Reprod.*, **4**, 140–146.